# The Dietary Protein/Carbohydrate Ratio Differentially Modifies Lipogenesis and Protein Synthesis in the Mammary Gland, Liver and Adipose Tissue during Gestation and Lactation

**DOI:** 10.1371/journal.pone.0069338

**Published:** 2013-07-16

**Authors:** Laura A. Velázquez-Villegas, Armando R. Tovar, Adriana M. López-Barradas, Nimbe Torres

**Affiliations:** Departamento de Fisiología de la Nutrición, Instituto Nacional de Ciencias Médicas y Nutrición, México, D.F., México; University of Santiago de Compostela School of Medicine - CIMUS, Spain

## Abstract

During gestation and lactation, a series of metabolic changes that are affected by the diet occurs in various organs of the mother. However, little is known about how the dietary protein (DP)/carbohydrate (DCH) ratio regulates the expression of metabolic genes in the mother. Therefore, the purpose of this work was to study the effect of consuming different percentages of DP/DCH, specifically 10/73, 20/63 and 30/53%, on the expression of genes involved in lipogenesis and protein synthesis in the mammary gland, liver and adipose tissue during gestation and lactation in dams. While the amount of weight gained during gestation was similar for all groups, only dams fed with 30/53% DP/DCH maintained their weight during lactation. In the mammary gland, the expression of the genes involved in lipogenesis, specifically SREBP1 and FAS, was dramatically increased, and the expression of the genes involved in protein synthesis, such as mTOR1, and the phosphorylation of its target protein, S6K, were also increased throughout pregnancy and lactation, regardless of the concentration of DP/DCH. In the liver and adipose tissue, the expression of the genes and proteins involved in lipid metabolism was dependent on the proportion of DP/DCH. The consumption of a low-protein/high-carbohydrate diet increased the expression of lipogenic genes in the liver and adipose tissue and the amount of lipid deposition in the liver. Conversely, the consumption of a high-protein/low-carbohydrate diet increased the expression of genes involved in amino acid oxidation in the liver during gestation. The metabolic adaptations reflected by the changes in the expression of metabolic genes indicate that the mammary gland has a priority for milk synthesis, whereas the adaptations in the liver and adipose tissue are responsible for providing nutrients to the mammary gland to sustain milk synthesis.

## Introduction

Several morphological and physiological changes occur in the mammary gland during the processes of gestation and lactation [1]–[4]. During gestation, there is an increase in the formation of the epithelial cells, which are involved in milk synthesis, from mammary fat cells [5]. During this stage, several hormones are involved in the regulation of the expression of specific genes to prepare the mammary gland for a successful lactation period [6]–[12]. During lactation, there is a sharp increase in the synthesis of the components of the milk, particularly proteins, lipids and lactose, in the epithelial cells in the mammary gland [13].

To sustain all the metabolic adaptations that occur during gestation and lactation in the mammary gland, the supply of nutrients to the dam is essential. It has been demonstrated that food restriction during these periods can modify the outcome of lactation. Food deprivation or the consumption of a low-energy diet during gestation and lactation has been shown to reduce or stop milk synthesis and secretion [14]–[16]. Therefore, the amount and quality of the diet have profound effects on milk synthesis [17]. In addition to the diet, during short periods of time, the body organs of the dam can also supply nutrients to the mammary gland for the differentiation of cells during gestation, mainly for the synthesis of milk components during lactation. It is known that the liver and the adipose tissue can actively participate in the supply of nutrients to the mammary gland [18]–[22].

To prepare the mammary gland for gestation and lactation, it is known that the regulation of the expression of genes coding for the transcription factors and enzymes involved in anabolic and catabolic processes is required [13], [23]–[27]. In particular, these include genes involved in lipogenesis (SREBP-1c and fatty acid synthase FAS) [28], protein synthesis (mTOR) [29], glyceroneogenesis (PEPCK) [30], and fatty acid oxidation (CPT-1) [26]. In addition, the supply of nutrients may also regulate the phosphorylation state of proteins involved in the activation of protein synthesis (S6K) via mTOR [31], [32] and the energy status of the cell via adenosine monophosphate kinase (AMPK) [33], [34]. The correct activation of these pathways leads to optimal milk synthesis and secretion. This has been confirmed by numerous studies that have demonstrated that these and other genes are actively regulated during the gestation and lactation stages [13], [23]–[27]. In addition, the expression of several of these genes is regulated in a different manner in the liver, adipose tissue and mammary gland [19], [21], [22].

However, there is scarce evidence regarding whether the proportion of dietary protein (DP) and dietary carbohydrates (DCH) can regulate the expression, protein abundance and the phosphorylation state of proteins involved in the metabolic adaptations that take place in the mammary gland, the liver and adipose tissue and their subsequent effects on protein synthesis and lipid and amino acid metabolism during gestation and lactation. Thus, the aim of this work was to study these adaptations in the mammary gland, liver and adipose tissue of dams fed diets containing different percentages of DP/DCH, 10/73%, which does not meet the protein requirement, 20/63%, which constitutes an adequate protein requirement [35], or 30/53%, which is a high-protein diet that contains an excess of amino acids, during gestation and lactation. Our results demonstrate that the regulation of some processes are responsive to the proportion of DP/DCH, particularly in the liver and adipose tissue, whereas the mammary gland is an anabolic organ that expresses most of the metabolic genes in a similar fashion, independent of the DP/DCH ratio.

## Materials and Methods

### Animals

Female Wistar rats weighing 200 to 250 g were obtained from the animal research facility at the Instituto Nacional de Ciencias Médicas y Nutrición. The animals were housed in individual stainless steel cages at 18°C with a 12∶12 h light-dark cycle. The animals were allowed free access to water and low-protein/high-carbohydrate (10/73%), normal-protein/normal-carbohydrate (20/63%) or high-protein/low-carbohydrate (30/53%) diets (**Table 1**). Gestational age was determined by vaginal smear to detect spermatozoa. Adipose tissue, liver and mammary gland tissues were obtained from virgin rats, rats that had been pregnant for 5, 14 and 20 days, and rats that had been lactating for 5 and 12 days. After normal pregnancy and delivery, the litter size was adjusted to 8 pups per dam. The food intake of the dam was recorded daily, and the dams and pups were weighed every 3 days. Non-pregnant, non-lactating rats were used as control and were killed in their diestrus 2 period, to have no relevant hormonal changes between animals inside the group and are referred to as “virgin rats.” This study was approved by the Animal Care Committee of the Instituto Nacional de Ciencias Médicas y Nutrición, México, in accordance with international guidelines for the use of animals in research.

**Table 1:** Composition of the experimental diets used in this study.

Ingredients	Percentage (%) of dietary protein/dietary carbohydrates		
g/kg diet	10/73	20/63	30/53
Casein^a^	100.0	200.0	300.0
Dextrose^a^	152.5	132.0	107.3
Sucrose	115.3	100.0	100.0
Starch^a^	460.0	397.5	322.1
Soy oil	70.0	70.0	70.0
Mineral mix^b^	35.0	35.0	35.0
Vitamin mix^c^	10.0	10.0	10.0
L-cystine	3.0	3.0	3.0
Choline citrate	2.5	2.5	2.5
Cellulose	50.0	50.0	50.0

a Teklad test diets.

b Rogers-Harper, Teklad test diets.

c Vitamin mix, Teklad 40060 (milligrams per kilogram diet): p-aminobenzoic acid, 110; ascorbic acid, 991; biotin, 0.4; vitamin B12, 30; calcium pantothenate, 66; choline dihydrogen citrate, 3497; folic acid, 2; inositol, 110; menadione, 50; niacin, 99; pyridoxine HCl, 22; riboflavin, 22; thiamin HCl, 22; vitamin A palmitate, 40; cholecalciferol, 4; and vitamin.

### Quantitative Real-Time PCR

Total RNA was extracted from mammary gland, liver and adipose tissue by the guanidinium thiocyanate-cesium chloride method [36]. The RNA concentrations were measured using a Nanodrop spectrophotometer 1000 (ND-1000; Thermo Scientific, Wilmington, DE, USA). RNA (3 µg) was reverse-transcribed to cDNA by the use of Moloney murine leukemia virus (MMLV) reverse transcriptase (Invitrogen). For the real-time quantitative PCR analyses, 300 ng of cDNA was used in a final reaction volume of 10 µl per reaction. Predesigned TaqMan Assay (Applied Biosystems, Foster City, CA, USA) probes for fatty acid synthase (FAS Rn00569117_m1), hormone-sensitive lipase (HSL Rn00689222_m1), mechanistic target of rapamycin (mTOR Rn00571541_m1), phosphoenolpyruvate carboxykinase (PEPCK Rn01529014_m1), pyruvate kinase (PK1 Rn00561764_m1), serine dehydratase (SDH Rn00588631_m1), and sterol regulatory element binding protein 1 (SREBP1 Rn01495769_m1) were used. The Taqman Universal Master Mix was also provided by Applied Biosystems. The PCR scheme used was 48°C for 30 min, 95°C for 10 min, and then 40 cycles of 95°C for 15 sec and 60°C for 1 min. The amplification and detection of specific products was performed with the ABI PRISM 7000 (Applied Biosystems). The mRNA levels of the genes analyzed were normalized to the HPRT (hypoxanthine phosphoribosyltransferase 1) or beta-actin genes using the TaqMan probes Rn01527840_m1 and Rn00667869_m1, respectively, which were also obtained from Applied Biosystems. The relative amounts of all mRNAs were calculated using the comparative CT method (User Bulletin no. 2; PE Applied Biosystems).

### Western Blot

Proteins were extracted from the tissues using RIPA (Radio-Immunoprecipitation Assay) Lysis Buffer containing the following: 50 mM Tris-Cl, pH 7.4, 150 mM NaCl, 1% NP40, 0.25% Na-deoxycholate, and 1 mM PMSF. We added 1x Roche complete mini protease inhibitors. The protein concentration was measured in duplicate using the Bradford method. Before being loaded, the samples were prepared by mixing 40 µg of protein with Laemmli buffer in a 1∶1 ratio and heated at 80°C for 5 min.

The proteins were separated by electrophoresis on a polyacrylamide gel (8%), and transferred onto a polyvinylidene difluoride (PVDF) membrane (Amersham GE Healthcare). For gel electrophoresis and semi-dry transfer, we used a Tris/Glycine buffer. After transfer, the membranes were blocked with 5% non-fat milk blocking solution with 1x Tris-buffered saline (TBS) and 0.1% Tween 20 for one hour. The membranes were then incubated overnight at 4°C with the different primary antibodies as follows: FAS (Santa Cruz Biotechnology, sc-20140, 1∶2000), p70 S6 kinase α (Santa Cruz Biotechnology, sc-230, 1∶1000), p-p70 S6 kinase α (Santa Cruz Biotechnology, sc-11759, 1∶500), AMPK α1/2 (Santa Cruz Biotechnology, sc-25792, 1∶1000), P-AMPK α1/2 (Santa Cruz Biotechnology, sc-33524, 1∶500), and actin (Santa Cruz Biotechnology, sc-1615, 1∶1000), which were all diluted in 4 ml 1x TBS, 5% non-fat milk and 0.1% Tween 20. We used different secondary HRP (Horseradish peroxidase) conjugated antibodies as follows: anti-rabbit IgG-HRP (Santa Cruz Biotechnology, sc-2004, 1∶2000) and anti-goat IgG-HRP (Santa Cruz Biotechnology, sc-2768, 1∶2000) diluted in 4 ml 1x TBS, 5% non-fat milk and 0.1% Tween 20. The chemiluminescence produced was measured using Amersham Enhanced Chemiluminescence (ECL) detection reagents by exposure to X-ray film.

### Hematoxylin Eosin (H/E) Staining

The slides were deparaffinized at 70°C for 10 min. The tissue sections were rehydrated with two changes of xylene for 5 min each, xylene-alcohol (50–50), absolute alcohol and 96% alcohol. The sections were stained in a Harris hematoxylin solution for 8 min and washed in running tap water for 1 min. The slides were differentiated in 1% acid alcohol for 30 sec and washed in running tap water for 1 min, followed by an immersion in saturated lithium carbonate solution for 30 sec to 1 min. After being washed, the tissue sections were counterstained in eosin solution for 30 seconds to 1 min, dehydrated through 96% alcohol, absolute alcohol, xylene-alcohol (50–50) and xylene, and mounted with a xylene-based mounting medium.

## Statistical analysis

The results are reported as the means ± SEM. For biochemical parameters, the effect of time X % protein interaction was analyzed using a repeated-measures ANOVA to assess the main effects (Prism 5 for Mac OS X). The body weight and gene expression data were tested using a 1-way ANOVA, and significant differences among groups were analyzed by Bonferroni adjustments. Differences were considered significant at P<0.05.

## Results

### Serum Glucose and Insulin but no Leptin Concentrations during Gestation and Lactation are Modified by DP/DCH Ratio

During gestation and lactation, there were fluctuations in the serum glucose and insulin concentrations that depended of the DP/DCH ratio and the stage of gestation and lactation. This pattern was very irregular and did not show a specific trend for either gestation or lactation. On the other hand, only serum leptin concentration showed a significant change with the stage of gestation or lactation. During gestation, rats fed 10/73, 20/63 and 30/53% DP/DCH showed normal serum leptin concentrations that were increasing and reaching a peak at gestation day 20 (10 µg/L), subsequently leptin values decreased during lactation returning to normal concentrations. However, the serum leptin concentration was not significantly affected by the proportion of DP/DCH consumed by the dams (data no shown).

### The Proportion of Dietary Protein/Dietary Carbohydrate Affects the Weight Gain of Dams during Lactation and the Weight Gain of Offspring

**Figure 1:**
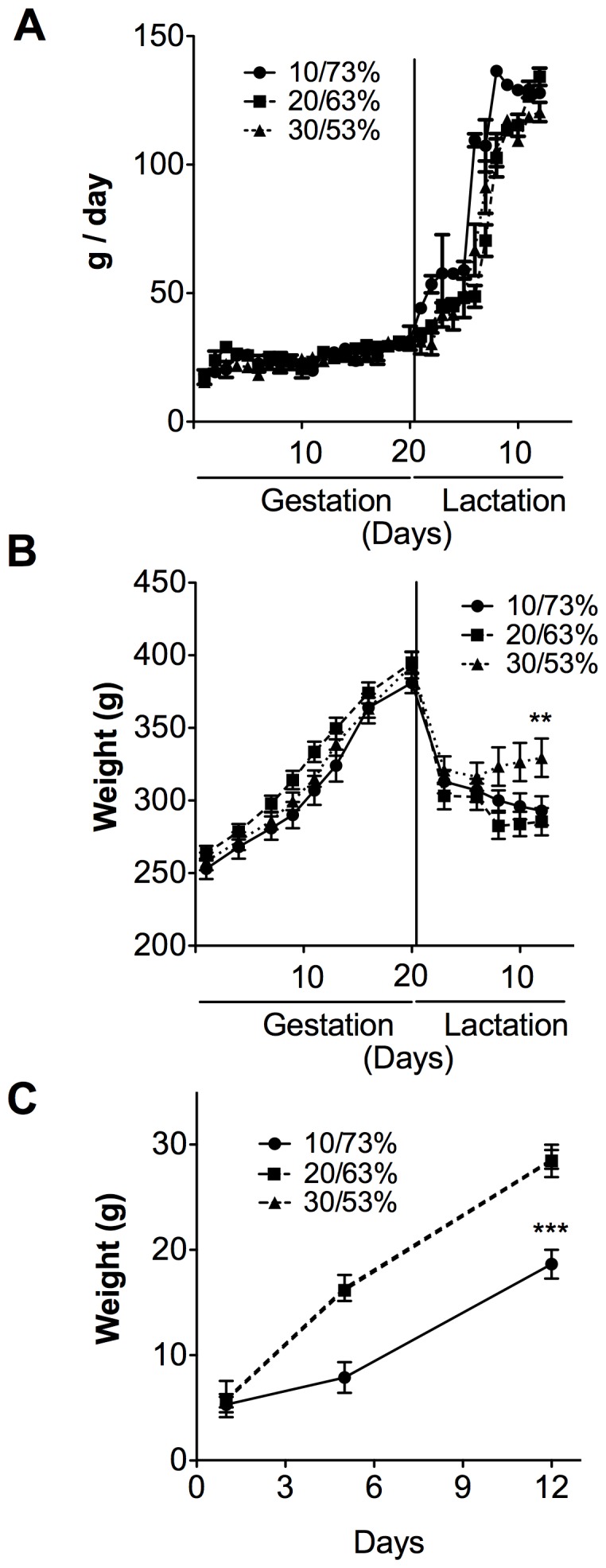
Food intake, body weight of dams fed different proportions (10/73, 20/63 or 30/53%) of dietary protein/dietary carbohydrate (DP/DCH) during gestation and lactation, as well as the pups’ weight gain. (A) The food intake, (B) weight gain of dams and (C) weight gain of pups from dams fed different proportions of DP/DCH (10/73, 20/63 or 30/53%) during gestation and lactation. The Values are mean ± SEM. n = 5. **p<0.01,***p<0.001.

To study the effect of the proportion of DP/DCH on the metabolic adaptations in the dams, food intake and weight gain were monitored during pregnancy and lactation. Food intake consumed during gestation was not significantly different among the groups (**Fig. 1a**), although there was a significant difference in the amount of DP/DCH consumed. There were no significant differences in weight gain among the groups fed 10/73, 20/63 and 30/53% DP/DCH during gestation, even on the last day of this period (**Fig. 1b**). These data suggest that the proportion of DP/DCH consumed with either diet was sufficient to sustain the weight gain of the dam during pregnancy (**Fig. 1b**). There was no difference in the body weights of the pups from any dietary group (**Fig. 1c**). However, pups from rats fed 10/73% DP/DCH gained significantly less weight than those fed 20/63 or 30/53% DP/DCH (**Fig. 1c**). Although the dams were consuming 30/53% DP/DCH, which provides an excess of amino acids, the pups gained the same amount of weight compared to pups from dams fed 20/63% DP/DCH. During the lactation period, the difference in the amount of DP had an impact on the dams’ and pups’ body weight. Rats fed 10/73 or 20/63% DP/DCH continued losing body weight during lactation, whereas dams fed 30/53% DP/DCH maintained their body weight (**Fig. 1b**) (p<0.01). This difference in body weight between the groups during lactation was associated with the amount of protein ingested. By day 12 of lactation, which corresponds to the peak of milk production, the dams fed 10/73, 20/63, and 30/53% DP/DCH consumed 9.98±1.06, 16.13±2.27, and 24.31±3.23 g protein/d, respectively.

### The Phosphorylation Status of S6K and AMPK is Modulated during Gestation and Lactation

**Figure 2:**
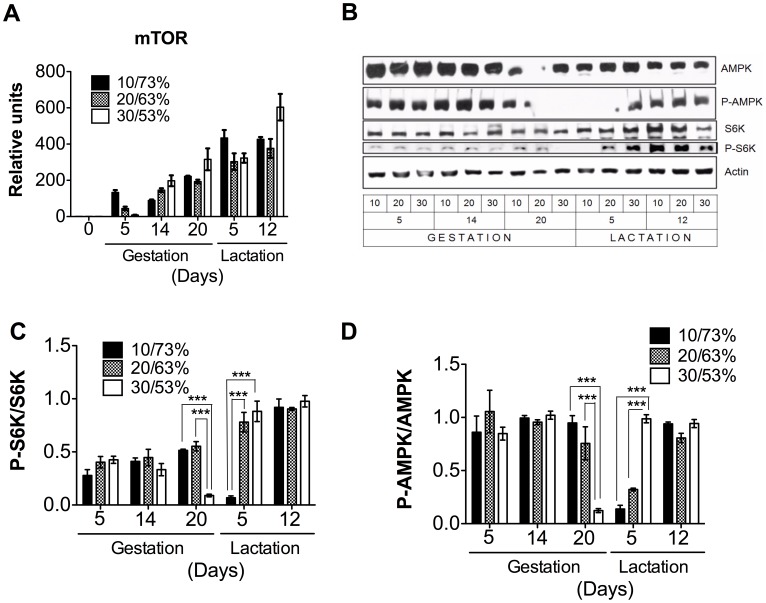
The genes and proteins involved in protein synthesis in the mammary gland of dams fed different proportions (10/73, 20/63 or 30/53%) of dietary protein/dietary carbohydrates (DP/DCH) during gestation and lactation. (A) The relative mRNA levels of mechanistic target of rapamycin (mTOR). (B) A representative immunoblot of AMP-activated protein kinase (AMPK), threonine phosphorylation of AMP-activated protein kinase (P-AMPK), p70 S6 kinase (S6K), threonine phosphorylation of S6 kinase (P-S6K), and actin. Western blot densitometric analysis of (C) P-S6K/S6K. (D) P-AMPK/AMPK. Values are the mean ± SEM of three different blots. n = 5. ***p<0.001.

We were interested in studying how the DP/DCH ratio could affect the expression of the key enzyme involved in protein synthesis, mTOR, in the mammary gland. We found that mTOR mRNA levels (**Fig. 2a**) showed a steady increase from gestation to lactation. Interestingly, despite the differences in the proportion of DP/DCH consumed, there was no significant difference among the groups in both stages. In addition, we measured the level of phosphorylation of S6K, a target protein of mTOR. We found that the phosphorylation levels of S6K significantly decreased in the transition from gestation to lactation, and it depended on the proportion of DP/DCH, the higher the amount of DP the sooner the decrease in S6K phosphorylation. We do not have a clear explanation for why the phosphorylation state of S6K in the mammary gland of rats fed 20/63% DP/DCH did not change. Nonetheless, at the peak of lactation, the levels of phosphorylated S6K was independent of the proportion of DP/DCH and was at its highest level, indicating, as expected, that the rate of protein synthesis was highest during this period (**Fig. 2b, c**). Furthermore, we examined whether the energy status of the mammary gland changed during gestation or lactation in response to the proportion of DP/DCH consumed. Thus, we measured the phosphorylation state of the AMPK. Interestingly, the phosphorylation status of AMPK was quite constant during both periods, with a sharp decrease around delivery. However, by day 12 of lactation, the phosphorylation status of AMPK reached a steady level, indicating a plateau in the energy state of the mammary gland (**Fig. 2b, d**). This is in agreement with previous studies that have shown that the phosphorylation of AMPK, which supports anabolic processes such as lipogenesis and lactose synthesis, is not increased during lactation [**34**].

### The Expression of Lipogenic Genes is not Affected by the Proportion of DP/DCH during Lactation but PEPCK Expression in the Mammary Gland is Modified

**Figure 3:**
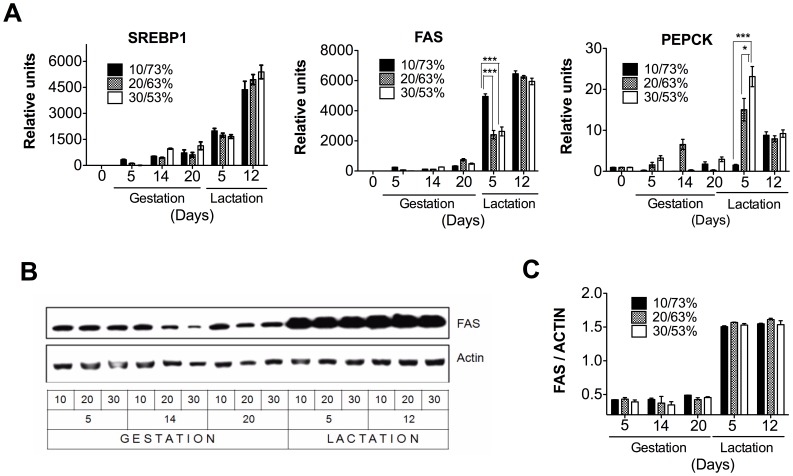
The genes and proteins involved in lipogenesis in the mammary gland of dams fed different proportions (10/73, 20/63 or 30/53%) of dietary protein/dietary carbohydrate (DP/DCH) during gestation and lactation. (A) The relative mRNA levels of sterol regulatory element binding protein 1 (SREBP1), fatty acid synthase (FAS), and phosphoenolpyruvate carboxykinase (PEPCK). (B) Representative immunoblot of FAS and actin. (C) Western blot densitometric analysis of FAS/ACTIN. Values are the mean ± SEM of three different blots. n = 5. *p<0.05, ***p<0.001.

Our data (**Fig. 3a, b, c**) clearly show the importance of the regulation of lipogenesis in the mammary gland. During gestation, the expression of the sterol regulatory element binding protein 1 (SREBP1), a key transcription factor involved in fatty acids biosynthesis, was slightly increased. Conversely, during lactation, SREBP1 was dramatically increased by 5000 fold (**Fig. 3a**). Fatty acid synthase (FAS), the target gene of SREBP1, also showed a dramatic increase in mRNA and protein abundance during lactation (**Fig. 3a, b, c**). Although we find a significant increase in FAS mRNA levels in the 10/73% DP/DCH group, we did not find significant differences in protein abundance among the three groups indicating that the response was independent of the proportion of DP/DCH. The highest expression of these lipogenic genes occurred at day 12, during the peak of lactation in which we did not find significant difference in mRNA levels and protein abundance among the three groups. Interestingly, we observed significant differences in the expression of phosphoenolpyruvate carboxykinase (PEPCK) on day 5 of lactation as the amount of DP increased (**Fig. 3a**).

### An Excess of DP Increased CPT-1 mRNA Expression in the Mammary Gland only during Gestation

**Figure 4:**
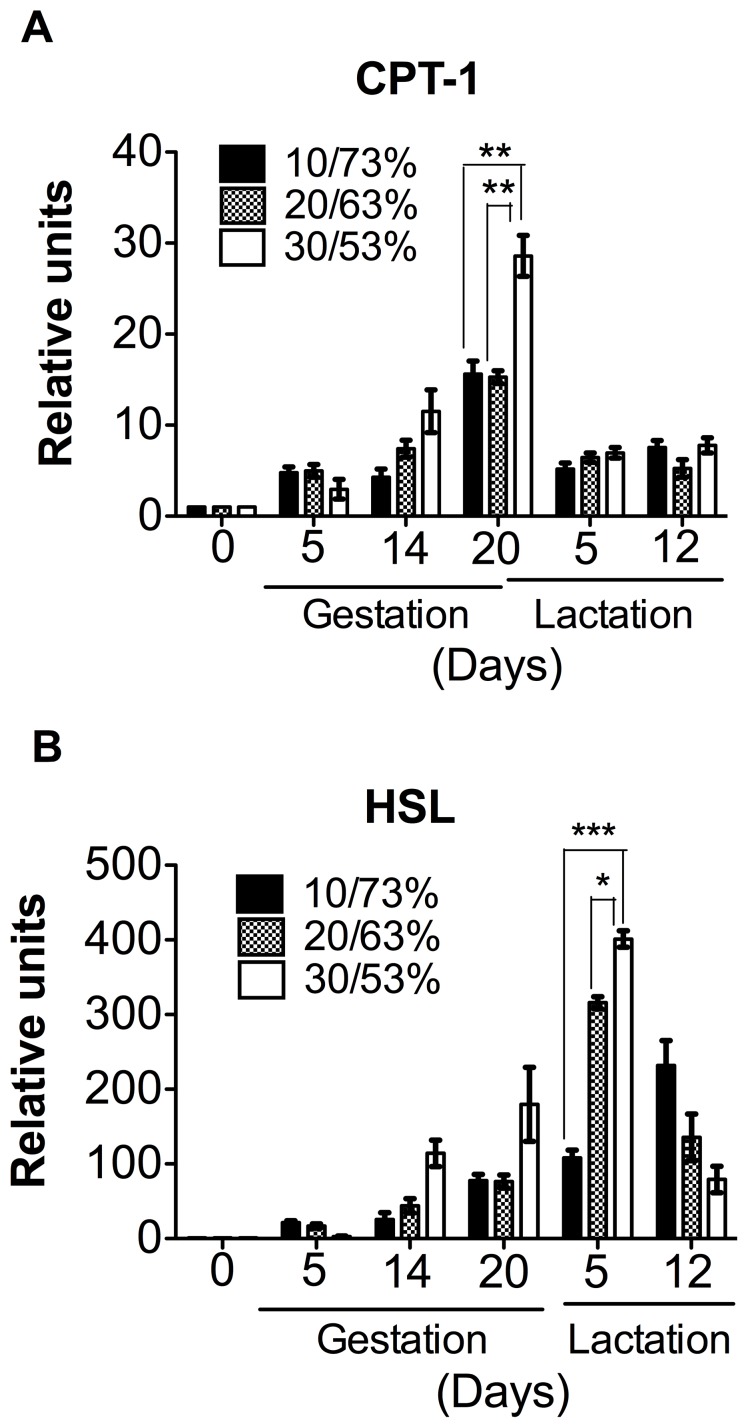
The expression of genes involved in lipid oxidation and lipolysis in the mammary gland of dams fed different proportions (10/73, 20/63 or 30/53%) of dietary protein/dietary carbohydrates (DP/DCH) during gestation and lactation. (A) The relative mRNA levels of carnitine palmitoyl transferase 1 (CPT-1) and (B) hormone sensitive lipase (HSL). Values are the mean ± SEM. n = 5. *p<0.05, **p<0.01, ***p<0.001.

We then explored whether the consumption of different proportions of DP/DCH could change the expression of genes involved in fatty acid oxidation. Our data showed that the expression of carnitine palmitoyl transferase-1 (CPT-1) showed a gradual increase during gestation, reaching a peak at delivery in the three different experimental groups (**Fig. 4a**). However, rats fed 30/53% DP/DCH showed a significant increase in the expression of CPT-1 with respect to groups fed 10/73 or 20/63% DP/DCH. After delivery, CPT-1 expression rapidly decreased at day 5 of the lactation period (**Fig. 4a**), indicating that a high-protein/low-carbohydrate diet (30/53% DP/DCH) exceeds the amino acid requirements during gestation but not during lactation. In addition, the expression of hormone-sensitive lipase (HSL) significantly increased during gestation in the rats fed 30/53% DP/DCH reaching its maximal peak of expression on day 5 of lactation and then rapidly decreased on day 12 of lactation (**Fig. 4b**).

### Expression of Genes Involved in Lipogenesis and Protein Synthesis in the Liver and Adipose Tissue did not Increase in a Similar Manner to the Mammary Gland

**Figure 5:**
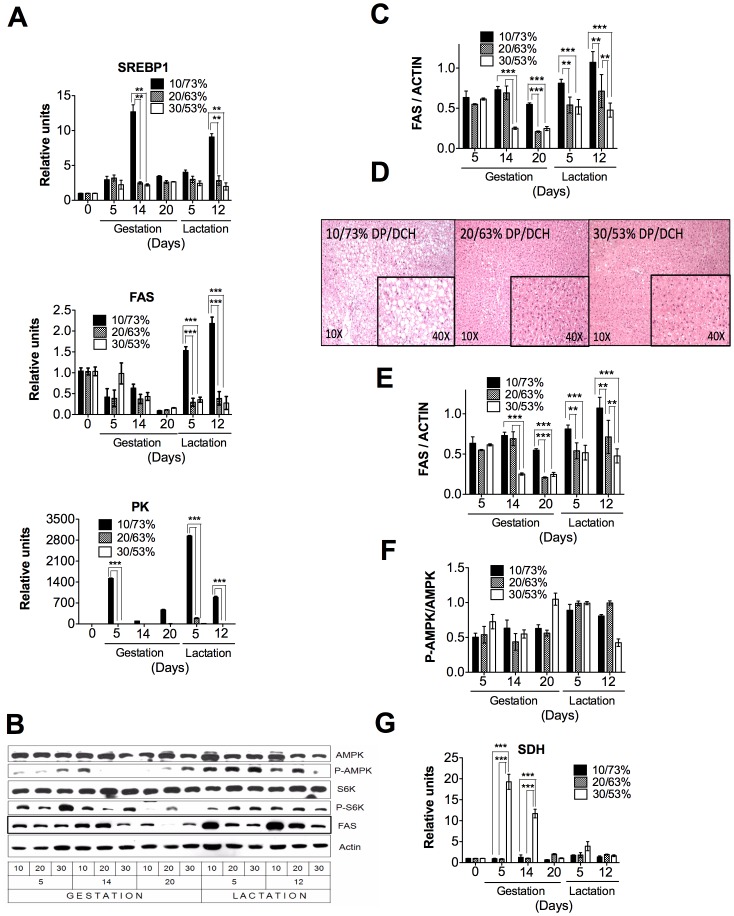
The expression of metabolic genes in the liver of dams fed different proportions (10/73, 20/63 or 30/53%) of dietary protein/dietary carbohydrates (DP/DCH) during gestation and lactation. (A) The relative mRNA levels of sterol regulatory element binding protein 1 (SREBP1), fatty acid synthase (FAS), and pyruvate kinase (PK). (B) A representative immunoblot of AMP-activated protein kinase (AMPK), threonine phosphorylation of AMP-activated protein kinase (P-AMPK), p70 S6 kinase (S6K), threonine phosphorylation of S6 kinase (P-S6K), fatty acid synthase (FAS) and actin. (C) Western blot densitometric analysis of FAS/ACTIN. (D) A representative histology picture of the liver of rats fed 10/73, 20/63 or 30/53% DP/DCH at day 5 of lactation. Western blot densitometric analysis of (E) P-S6K/S6K, (F) P-AMPK/AMPK. (G) The relative mRNA levels of serine dehydratase (SDH). Values are the mean ± SEM of three different blots. n = 5. **p<0.01, ***p<0.001.

We did not know whether these adaptations observed in the mammary gland occurred in other organs during the gestation and lactation periods. During gestation and lactation, the liver of rats showed a different pattern of expression of metabolic genes compared to the mammary gland. Glycolytic (PK) and lipogenic (FAS) enzymes and SREBP1 were highly expressed after the consumption of a low-protein diet, most likely due to the composition of the diet that was 10% protein and 73% carbohydrates. However, during the consumption of adequate proportion (20/63% DP/DCH) or high proportion of protein (30/53% DP/DCH) diets, the expression of these genes remained constant (**Fig. 5a**). Similarly, protein abundance of FAS was higher in the group fed 10/73% DP/DCH than those fed 20/63 or 30/53% DP/DCH (**Fig. 5b,c**) The elevated expression of lipogenic genes in the liver of rats fed 10/73% DP/DCH was associated with the development of fatty liver during lactation (**Fig. 5d**). S6K phosphorylation that is one of the target proteins of mTOR1 was largely unchanged during gestation and lactation by the different proportions of DP/DCH (**Fig. 5b, e**). The energy status of the liver cells, as represented by the phosphorylated AMPK (P-AMPK)/total AMPK ratio, was slightly increased during lactation (**Fig. 5b, f**). During gestation, but not during lactation, the expression of the amino acid degrading enzymes, such as serine dehydratase (SDH), was significantly increased only when rats consumed a high protein diet in the group of 30/53% DP/DCH diet (**Fig. 5g**).

**Figure 6:**
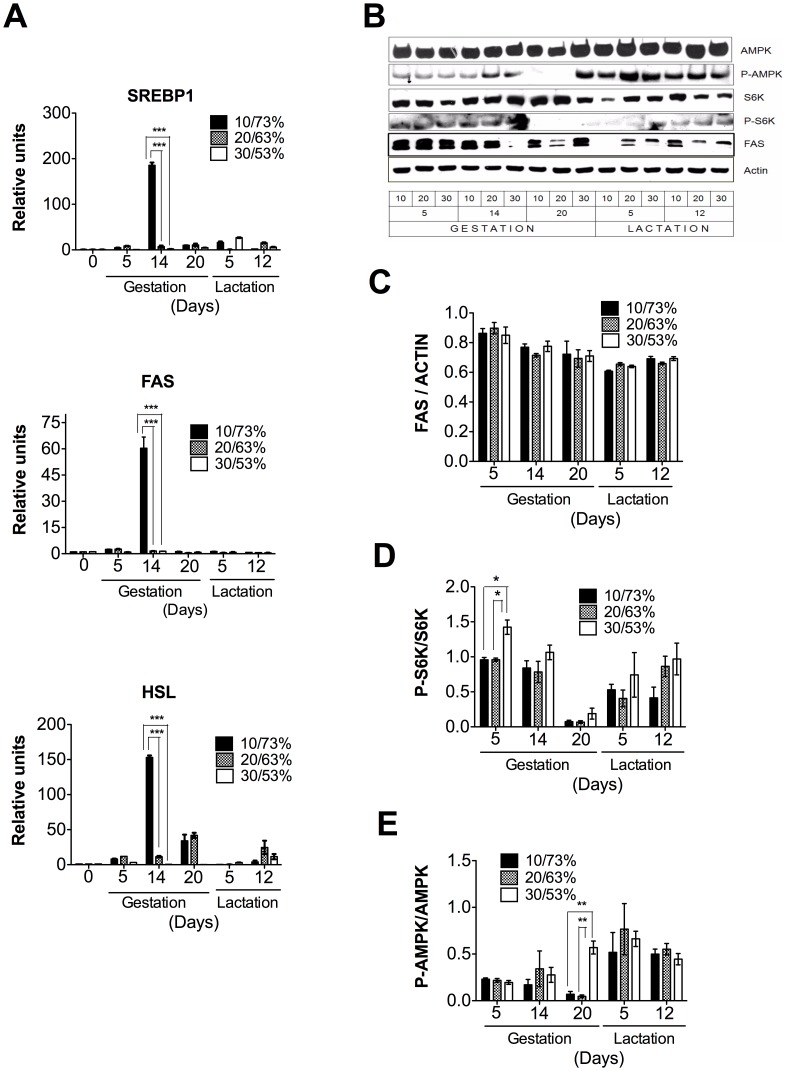
The expression of metabolic genes in the adipose tissue of dams fed different proportions (10/73, 20/63 or 30/53%) of dietary protein/dietary carbohydrates (DP/DCH) during gestation and lactation. (A) The relative mRNA levels of sterol regulatory element binding protein 1 (SREBP1), fatty acid synthase (FAS), and hormone sensitive lipase (HSL). (B) A representative immunoblot of AMP-activated protein kinase (AMPK), threonine phosphorylation of AMP-activated protein kinase (P-AMPK), p70 S6 kinase (S6K), threonine phosphorylation of S6 kinase (P-S6K), fatty acid synthase (FAS) and actin. Western blot densitometric analysis of (C) FAS/ACTIN, (D) P-S6K/S6K and (E) P-AMPK/AMPK. Values are the mean ± SEM of three different blots. n = 5. *p<0.05, **p<0.01 ***p<0.001.

The metabolic adaptations that occur during gestation and lactation in adipose tissue were very similar to those observed in the liver. The expression of lipogenic genes, as well as that of HSL, only increased when rats consumed a low-protein/high-carbohydrate diet (**Fig. 6a**). In fact, FAS abundance decreased with the progression of gestation and lactation (**Fig. 6b, c**). During gestation, S6K is fully active after phosphorylation at Thr^389^. At delivery, the phosphorylation of S6K almost disappeared but rapidly increased again during the lactation period, reaching values similar to those observed during gestation (**Fig. 6b, d**). During the gestation period, AMPK is partially activated by phosphorylation at Thr^172^, but this phosphorylation decreased rapidly during delivery and fully restored during lactation (**Fig. 6b, e**).

## Discussion

Several studies have shown that nutritionally adverse conditions during pregnancy can lead to physiologic adaptations, including changes in gene expression, to cope with the limited availability of amino acids. However, little is known about the metabolic adaptations that occur in the different organs of the mother during pregnancy and lactation under different proportions of DP/DCH.

One of the most profound adaptations is in energy homeostasis, where the mother faces the competing needs of meeting her own energy demands, supplying nutrients to the growing fetus, and also establishing a positive energy balance to promote the storage of energy resources in preparation for the large metabolic demands of lactation. The results in the present study show that only dams fed 30/53% DP/DCH have a prompt recovery in their body weight at day 12 of lactation.

In mammary tissue, the onset of gestation promotes the activation of anabolic processes, such as protein synthesis mediated by mTOR1 and the phosphorylation of its target protein S6K, which are barely affected by the DP/DCH ratio, however, it is clear that a low protein 10% DP even when energy is compensated with carbohydrates was not enough to sustain body weight of the dam during lactation.

The onset of the lactation period dramatically increases the expression of the lipogenic enzyme FAS mediated by the transcription factor SREBP1, and reduces the expression of the CPT-1 involved in fatty acid oxidation. These results suggest that there is a significant supply of fatty acid to form triglycerides for milk production that will help the newborn gain energy and maintain body temperature [37]. The increase in the expression of lipogenic genes in this tissue was not affected by the proportion of DP/DCH.

The lactating mammary gland is one of the most active triglyceride-synthesizing organs in the body and requires glyceroneogenesis mediated by PEPCK [30]. In the mammary gland, PEPCK may contribute to the milk content by providing the backbone for triglyceride synthesis [30]. Interestingly, our data showed that during lactation, PEPCK expression depends on the DP concentration, which suggests that although the fatty acid synthesis is not affected by the DP/DCH ratio (**Fig. 3a**), fatty acid esterification may be the limiting step in rats fed the 10% casein diet due to a reduction in the production of glycerol. This, in turn, may result in a decrease in triglyceride formation and therefore, affect the growth rate of the offspring (**Fig. 1a** **).**

In contrast, during gestation, we observed a gradual increase in the expression of CPT-1 in the mammary gland reaching a peak of expression immediately before birth, when estradiol levels are the lowest [38]. This suggest that estradiol levels during pregnancy suppress the expression of genes involved in lipid oxidation and promote those of triglyceride synthesis in the mammary gland. However, immediately before birth, the decrease in estradiol levels is associated with a concomitant increase in CPT-1 mRNA expression. Interestingly, our data showed that a high-protein/low-carbohydrate diet results in a more significant CPT-1 mRNA abundance in the mammary gland, (**Fig. 4a** **)**. In addition, the *in silico* analysis of the rat CPT-1 promoter region that we performed with the MatInspector program for transcription factor binding sites (unpublished observations) showed several putative estrogen response elements (ERE), suggesting that estrogens may directly regulate the transcription of CPT-1. During lactation, there is a decrease in the expression of CPT-1 and these changes are related to the sharp increase in mammary gland lipogenesis, which is needed to synthesize large amounts of triglycerides for milk production. Our results are in agreement with a previous microarray analysis study that reported a decrease in the expression of enzymes of fatty acid oxidation during lactation [26], [37].

Interestingly, in the mammary gland, we also observed a steady increase in the expression of hormone-sensitive lipase (HSL) from gestation to lactation, which is important for the hydrolysis of triglycerides in the intestine of the newborn, because HSL is secreted into the milk [39]. Our study also showed that the increase of HSL depended also of the proportion of DP/DCH consumed.

The AMPK has emerged as a key nutrient sensor with the ability to regulate whole-body metabolism. Upon activation, AMPK turns on catabolic pathways to restore ATP levels. However, in situations such as gestation, birth and lactation, fatty acid oxidation and lipogenesis seem to be independent of AMPK regulation in the mammary gland, which is in an accelerated anabolic state.

In contrast to the response observed in the mammary gland, it has been reported that other organs of the dam show metabolic adaptations in response to periods of restriction of specific nutrients. In the liver during gestation, the hepatic amino acid degrading enzymes, such as SDH, rapidly increased when rats consumed a high-protein/low-carbohydrate diet to oxidize the excess of amino acids, however, during lactation when the amino acid requirement increases, the oxidation of amino acids decreases to conserve body nitrogen.

Our results also showed that a low-protein/high-carbohydrate diet during pregnancy favors the synthesis of lipids in the liver and adipose tissue, as evidenced by an increase in the expression of SREBP1 and FAS. During lactation, the expression of SREBP1, FAS and PK genes, as well as the protein abundance of FAS, remained elevated in the livers of dams fed a low-protein/high-carbohydrate diet. This favors lipid accumulation, as observed in the histological analysis of the liver on day 5 of lactation. This response may be due to the excess of carbohydrates in the diet, which activates lipogenesis. In addition, the deficiency of amino acids might reduce Apoprotein synthesis limiting the export of triglycerides into the circulation. This leads to the accumulation of triglycerides caused by the abnormal secretion of very low-density lipoproteins (VLDL) [40], producing a fatty liver.

In adipose tissue, there was a decrease in the expression of FAS and SREBP-1 during lactation (**Fig. 6a**). During lactation, the expression of lipoprotein lipase (LPL) increased in the mammary gland and decreased in adipose tissue [41]. LPL hydrolyzes chylomicrons and VLDL to remove triglycerides from the circulation and provide fatty acids to different tissues. The net result of the changes of LPL activity during lactation is that the triglycerides available in the circulation are directed to the mammary gland, rather than to adipose tissue to support milk production [42].

Although both the liver and the adipose tissue are capable of de novo fatty acid synthesis, the mammary gland is the main producer of fatty acids in this physiological period [26].

Additionally, the pattern of phosphorylation of AMPK is increased during lactation in this tissue. These results suggest that the role of adipose tissue in this period is to activate the process of lipolysis in order to provide fatty acids to the mammary gland for the synthesis of triglycerides in milk.

In conclusion, although the mammary gland performs a metabolic “switch” in the transition from gestation to lactation, regardless of the DP/DCH ratio, other organs, such as the liver and adipose tissue, do respond to the different proportions of DP/DCH consumed and make the necessary adaptations to supply nutrients to the mammary gland to sustain milk synthesis during lactation.
